# Increased airway epithelial cell–derived exosomes activate macrophage‐mediated allergic inflammation via CD100 shedding

**DOI:** 10.1111/jcmm.16843

**Published:** 2021-08-20

**Authors:** Yi Yu, Yao Zhou, Caixia Di, Caiqi Zhao, Jie Chen, Wen Su, Qun Wu, Min Wu, Xiao Su, Zhenwei Xia

**Affiliations:** ^1^ Department of Pediatrics Ruijin Hospital Shanghai Jiao‐tong University School of Medicine Shanghai China; ^2^ Unit of Respiratory Infection and Immunity Institut Pasteur of Shanghai, Chinese Academy of Sciences Shanghai China; ^3^ School of Medicine and Health Sciences Department of Biomedical Sciences University of North Dakota Grand Forks ND USA

**Keywords:** asthma, CD100, exosomes, MMP14, PLXNB2

## Abstract

Airway epithelial cells (AECs) participate in allergic airway inflammation by producing mediators in response to allergen stimulation. Whether ovalbumin (OVA) challenge promotes exosome release from AECs (OVA‐challenged AEC‐derived exosomes (OAEs)), thereby affecting airway inflammation, as well as the underlying mechanisms, is unknown. Our study showed that AECs released an increased number of exosomes after OVA challenge, and the expression of Plexin B2 (PLXNB2; a natural CD100 ligand) was increased by a massive 85.7‐fold in OAEs than in PBS‐treated AEC‐derived exosomes (PAEs). CD100^+^F4/80^+^ macrophages engulfed OAEs to trigger the transcription of pro‐inflammatory chemokines and cytokines. *Plxnb2* transcripts increased in asthmatic lungs, and similarly, PLXNB2 protein was highly enriched in exosomes purified from asthmatic bronchoalveolar lavage (BAL) fluid. Furthermore, aspiration of PLXNB2 or OAEs increased the recruitment of lung neutrophils, monocytes, eosinophils and dendritic cells in OVA‐challenged mice. Mechanistically, OAE aspiration enhanced the cleavage of CD100 by MMP14, which manifested as an increase in the soluble CD100 (sCD100) level in BAL fluid and lung homogenates. Knockdown of *Mmp14* in macrophages prevented the cleavage of CD100 and reduced *Ccl2*, *Ccl5* and *Cxcl2* transcription. These data indicate that PLXNB2‐containing OAEs aggravate airway asthmatic inflammation via cleavage of CD100 by MMP14, suggesting potential therapeutic targets of OAE‐mediated asthma exacerbations.

## 1 INTRODUCTION

Airway hypersensitivity and inflammation during asthma involve not only immune cells but also airway epithelial cells (AECs). AEC‐secreted cytokines (ie IL‐25, IL‐33 and TLSP) are important communicators that regulate immune effector cells. Recent studies have indicated that epithelial‐derived exosomes contain various components, including cytokines, as communicators to modulate airway inflammation, including in asthma,^1^, ^2^, ^3^, ^4^ acute lung injury,^5^ idiopathic pulmonary fibrosis^6^ and bronchopulmonary dysplasia.^7^

Exosomes are extracellular nanovesicles that are usually 30 to 150 nm in size, that are secreted from various cell types and that are found in all mammalian tissues and fluids. They are lipid bilayer‐enclosed extracellular structures containing proteins and nucleic acids (DNAs, mRNA, miRNAs and other ncRNAs). These bioactive materials can be transferred from a donor cell to a recipient cell, modulating the latter's function. The quantity of exosomes is increased in the bronchoalveolar lavage (BAL) fluid of asthma patients and asthmatic animal models.^8^, ^9^ The roles of exosomes released by several immune cells are relatively well studied. Dendritic cell–derived exosomes can induce CD4+ T cell activation.^10^ Neutrophil‐derived exosomes can bind and degrade the extracellular matrix,^11^ enhance the proliferation of airway smooth muscle cells and promote airway remodelling during the progression of asthma.^12^ AECs are thought to be the main source of exosomes in BAL fluid. In the airways, exosomes released by AECs modulate the immune response to viral infection.^13^ Under the influence of IL‐13, epithelial cell–derived exosomes can induce enhanced proliferation and chemotaxis of undifferentiated macrophages in the lungs under asthmatic inflammatory conditions.^2^

Since ovalbumin (OVA) is frequently used to induce asthma, in this study, we hypothesized that OVA challenge would stimulate airway epithelial cells to produce exosomes (OVA‐challenged AEC‐derived exosomes (OAEs)) that affect airway inflammation. Therefore, the objectives were as follows: (a) to determine whether AECs produce OAEs in response to OVA challenge and what the main contents of the exosomes are; (b) to determine whether macrophages internalize OAEs and change transcription of pro‐inflammatory chemokines and cytokines; and (c) to determine whether sensitization with OAEs or their components augments airway inflammation in response to OVA challenge and what the underlying mechanisms are. We found that airway epithelial cell–derived exosomes (containing PLXNB2) were increased in number in response to OVA challenge. Macrophages engulf OAEs, which promote the transcription of pro‐inflammatory chemokines and cytokines. OAEs or PLXNB2 augmented airway inflammation by recruiting pro‐inflammatory cells in OVA‐challenged mice. Mechanistically, OAE aspiration increased the cleavage of CD100 by MMP14 in OVA‐challenged lungs. In OAE‐challenged macrophages, knockdown of *Mmp14* abolished the cleavage of CD100. These findings indicate that OVA‐challenged AECs produce PLXNB2‐containing exosomes to worsen airway inflammation via MMP14‐mediated cleavage of CD100.

## 2 MATERIALS AND METHODS

### 2.1 Cell culture

The human bronchial epithelial cell line BEAS‐2B (ATCC CRL‐96–6) was cultured in DMEM (Thermo Fisher Scientific) supplemented with 10% foetal bovine serum (FBS; Thermo Fisher Scientific), 100 U/ml penicillin and 100 mg/ml streptomycin (Thermo Fisher Scientific). When cells reached 70% confluence, they were washed twice with 5 ml of PBS (Thermo Fisher Scientific) and then incubated with ovalbumin (OVA; 1 mg/ml; Sigma‐Aldrich) in DMEM for 24‐h exosome isolation. Primary epithelial cells isolated and purified from mouse tracheas^14^ were seeded in 12‐well cell culture plates and cultured in growth factor–enriched medium (Procell). When cells reached 70% confluence, they were washed twice with 5 ml of PBS and incubated for 24 h in DMEM supplemented with 10% exosome‐free FBS containing 1 mg/ml OVA. Exosome‐free FBS was obtained by overnight (16 h) ultracentrifugation.^15^ RAW264.7 (ATCC TIB‐71) macrophages were cultured with DMEM (Thermo Fisher Scientific) supplemented with 10% FBS (Thermo Fisher Scientific), 100 U/ml penicillin and 100 mg/ml streptomycin (Thermo Fisher Scientific) and used for co‐culture with OAEs (10 ng/ml) for further analysis.

### 2.2 Exosome isolation and identification

Cell culture supernatant was collected and subjected to two centrifugations: 300 g for 10 min (to pellet floating cells) and 3000 g for 15 min (to pellet cell debris). Exosome pellets were collected by a series of ultracentrifugations^16^ at 10,000 g for 30 min and 100,000 g for 70 min. The pellets were washed once with PBS and subjected to another ultracentrifugation at 100,000 g for 70 min. For the BEAS‐2B cell line, exosomes isolated from 250 ml of conditioned medium were resuspended in 250 μl of PBS for in vivo experiments. For primary airway epithelial cells (pAECs), exosomes isolated from 24 ml of conditioned or unconditioned medium were resuspended in 100 μl of SDS (Beyotime) or PBS for proteomic or nanoparticle tracking analysis (NTA). The morphology and size of the isolated exosomes were visualized by transmission electron microscopy (TEM). Western blotting was used to identify exosomes containing the markers CD63 and Tsg101.

### 2.3 Western blot analysis

Total protein extracted from cells, exosomes or lung homogenates was prepared and solubilized in ice‐cold RIPA buffer (Beyotime) supplemented with a protease and phosphatase inhibitor cocktail (Thermo Fisher Scientific). Extracts containing 30–50 μg of protein were separated by 10% SDS‐PAGE and then transferred to PVDF membranes. The membranes were blocked with Tris‐buffered saline‐Tween‐20 buffer containing 5% skim milk and incubated with the following primary Abs: mouse anti‐CD63 antibody (Santa Cruz), mouse anti‐Tsg101 antibody (Santa Cruz), mouse anti‐Semaphorin‐4D/CD100 (E8S8A) mAb (CST), rabbit anti‐MMP14 polyclonal antibody (Absin), mouse anti‐Plexin B2 antibody (R&D) or rabbit anti‐β‐actin polyclonal antibody (CST). The samples were incubated overnight, followed by the addition of a corresponding anti‐rabbit, anti‐sheep (R&D) or anti‐mouse IgG secondary Ab (CST). Signals were detected via enhanced chemiluminescence using a Thermo ECL kit (Thermo Fisher Scientific).

### 2.4 Mass spectrometry analysis, protein quantification and proteomic analysis

Liquid chromatography‐tandem mass spectrometry was performed on a separate EASY nLC HPLC system (Thermo Fisher Scientific, nanoViper C18 and EASY column) coupled to a Q Exactive (Thermo Fisher Scientific) mass spectrometer. The iBAQ method was applied to quantify proteins. The iBAQ intensity approximates the absolute quantity of a protein. Differential protein expression in PAEs and OAEs was defined as a fold change (FC) ≥3. The FC was calculated as the iBAQ intensity of OAEs divided by that of PAEs. GraphPad software was used to generate a heat map. GO enrichment analysis was performed by an online service provided by BGI Genomics.

### 2.5 Mice

Female C57BL/6J mice (6–8 weeks old) were purchased from the Shanghai Laboratory Animal Center. Under specific pathogen–free conditions, mice were housed in groups with 12‐h dark/light cycles and free access to food and water. Anaesthesia was administered by intraperitoneal (i.p.) injection of pentobarbital sodium (50 mg/kg). All of the animal experiments were approved by the Institut Pasteur of Shanghai, Chinese Academy of Sciences.

### 2.6 Asthma models

OAEse (se: sensitization) + OVAc (c: challenge) model: Mice were intranasally (i.n.) sensitized with 25 μg of OAEs (10e9 exosomes in 50 μl of sterile PBS) on days 0, 3, 6 and 9; challenged with OVA (100 μg/ml) on days 11, 12 and 13; and killed on day 14. PBS‐treated and OVA‐challenged mice served as controls.

OAE‐treated OVA‐induced asthma model (asthma +OAEs): Mice were sensitized and challenged as described previously^17^ and divided into 3 groups. **Control group**: Mice were sensitized and challenged with PBS. **Asthma +PBS group**: Mice were sensitized with an i.p. injection of OVA (100 μg) in Al(OH)3 on days 0 and 14 and challenged with an aspiration of OVA (100 μg) on days 23, 24, and 25. At the designated time points, mice also received an aspiration of PBS. **Asthma +OAE group**: Mice were sensitized with an i.p. injection of OVA (100 μg) in Al(OH)3 on days 0 and 14 and challenged with an aspiration of OVA (100 μg) on days 23, 24 and 25. At the designated time points, mice also received an aspiration of OAEs.

### 2.7 Measurement of AHR

At 24 h after the final treatment, airway hyper‐responsiveness (AHR) was assayed using an AniRes2005 Lung Function System (version 3.5; Bestlab Technology Co., Ltd.). Mice were anaesthetized with 50 mg/kg pentobarbital sodium and connected to a computerized small animal ventilator via a tracheal cannula. The time of expiration/inspiration and the respiratory rate were preset at 2:1 and 90 breaths/min, respectively. The resistance of the lung (RL), resistance to expiration (Re) and respiratory dynamic compliance (Cdyn) were recorded to evaluate the reaction of mice to a methacholine chloride gradient (0.0125, 0.025, 0.05, mg/kg body weight). This compound was injected into the jugular vein at 5‐min intervals using a fine needle.^18^

### 2.8 BAL fluid collection and lung single‐cell preparation

For some experiments, BAL fluid was collected using 3 × 0.5 ml PBS. The tube was centrifuged at 500 g for 7 min at 4℃. The supernatant was then collected and stored at −80℃, and the pellet was resuspended in 100 μl FACS buffer for flow cytometry.

Mouse lungs were carefully perfused and incubated with collagenase type I (1 mg/mL; Sigma‐Aldrich Corp.) and type I bovine pancreatic DNase (20 μg/ml; Sigma‐Aldrich Corp.) for 45 min at 37℃ in PBS containing 2% FBS. After incubation, 10 ml of PBS was added to stop the reaction. Then, the digested lung tissue was passed through a 20‐G needle with a 5‐ml syringe 10–15 times to form a single‐cell suspension. The tube was centrifuged at 500 g for 5 min at 4℃, and the pellet was collected and resuspended in 2 ml of red blood cell lysis buffer, incubated at room temperature for 5 min and then washed with 10 ml of PBS at 500 g for 5 min at 4℃. The single lung cells were then ready for staining.

### 2.9 Depletion of alveolar macrophages

Alveolar macrophage (AM) depletion was achieved by intratracheal administration of 60 µl of clodronate liposomes and has been successfully used to study the role of alveolar macrophages in a variety of disease processes.^19^ Clodronate liposomes (Clophosome®—Clodronate Liposomes (Neutral), FormuMax Scientific Inc.) or control liposomes (Plain Control Liposomes for Clophosome® (Neutral), FormuMax Scientific Inc.) were purchased. A single dose of 60 µl of clodronate liposomes per mouse was found to result in a 90% reduction in the number of AMs, and a single dose resulted in sustained AM depletion for 7 days.

### 2.10 Adoptive transfer of macrophages

C57BL/6 mice were depleted of resident AMs by intratracheal administration of a single dose of clodronate liposomes 3 days before transfer. RAW264.7 cells or CD100 knockdown RAW264.7 cells were washed with PBS and then directly instilled (in 20 μl PBS) into the lungs of these mice at 3 days (2e5 cells per mouse). A dose of 25 µg of OAEs was also administered intranasally on the same day. Thirty‐six hours after adoptive macrophage transfer, BAL fluid and lung tissue were collected for further experiments.

### 2.11 Flow cytometric analysis (FACS), pulmonary eosinophil sorting and live microscopy

Purified rat anti‐mouse CD16/CD32, anti‐CD11c‐AF700, anti‐Gr‐1(Ly6C/Ly6G)‐PE (eBioscience), anti‐CD11b‐PerCP‐Cy5.5, anti‐F4/80‐APC (BioLegend), anti‐CD45.1‐APC‐Cy7/FITC, anti‐Ly6C‐BV421, anti‐Ly6G‐APC, anti‐Siglec‐F‐PE/FITC, anti‐MHCII‐PE/FITC, anti‐B220‐BV510, anti‐FVS‐BV605/BV510 (BD), anti‐Semaphorin‐4D/CD100 (E8S8A) rabbit monoclonal (CST), anti‐MMP14 rabbit polyclonal (Absin, CN), goat anti‐rabbit IgG H&L‐AF488 and goat anti‐rabbit IgG H&L‐AF594 (Abcam) antibodies were used. Experiments were performed on a Cytoflex or BD LSR Fortessa flow cytometer. Pulmonary polymorphonuclear cells were isolated from asthmatic mice using the Histopaque purification method (Histopaque 11191 and 10771; Sigma‐Aldrich), and then, eosinophils (siglec‐F^+^) were sorted by flow cytometry. After resting in a confocal dish for 30 min, 10 μl of OAEs was added on the edge of the dish, and the movement and morphology of the eosinophils were recorded by a live‐cell workstation (GE Delta vision).

### 2.12 ELISA

CCL2 and sCD100 levels in the BAL fluid and cell culture supernatant were determined with a CCL2 (BioLegend) ELISA kit and a mouse Semaphorin 4D/CD100 ELISA kit (Colorimetric) (Novus), respectively.

### 2.13 Quantitative RT‐PCR analysis

Total mRNA was isolated from lung homogenates or RAW264.7 cells using an RNeasy kit (QIAGEN). For reverse transcription, 1 μg of mRNA was used to generate cDNA, followed by quantitative PCR using SYBR Green (QIAGEN) to assess the transcription of *Ccl2*, *Ccl5*, *Cxcl2*, *Csf2*, *Il6*, *Tnf*‐*α*, *Il*‐*1β*, *Il*‐*12a*, *Il*‐*12b* and *Mmp*14. The primer sequences were as follows:

*Ccl2*: F 5′‐GAAGGAATGGGTCCAGACAT‐3′, R 5′‐ACGGGTCAACTTCACATTCA‐3′;

*Ccl5*: F 5′‐*GCTGCTTTGCCTACCTCTCC*‐3′, R 5′‐*TCGAGTGACAAACACGACTGC*‐3′;

*Cxcl2*: F 5′‐CCAACCACCAGGCTACAGG‐3′, R 5′‐GCGTCACACTCAAGCTCTG‐3′;

*Csf2*: F 5′‐ATGCCTGTCACGTTGAATGAAG−3′, R 5′‐GCGGGTCTGCACACATGTTA‐3′;

*Il6*: F 5’‐GGCCTTCCCTACTTCACAAG‐3’, R 5’‐ATTTCCACGATTTCCCAGAG‐3’;

Tnf‐α: F 5′‐ GGAACACGTCGTGGGATAATG−3′, R 5′‐ GGCAGACTTTGGATGCTTCTT‐3′;

Il‐1β: F 5′‐ CCAAAAGATGAAGGGCTGCT−3′, R 5′‐ ACAGAGGATGGGCTCTTCT −3′;

Il‐12a: F 5′‐CAATCACGCTACCTCCTCTTTT−3′, R 5′‐CAGCAGTGCAGGAATAATGTTTC‐3′;

Il‐12b: F 5′‐ GTCCTCAGAAGCTAACCATCTCC−3′, R 5′‐ CCAGAGCCTATGACTCCATGTC‐3′;

*Mmp14*: *F 5*′‐*CAGTATGGCTACCTACCTCCAG* −*3*′, *R 5*′‐*GCCTTGCCTGTCACTTGTAAA*‐*3*′;

*Gapdh*: 5′‐AGCCCAGGATGCCCTTTAGT‐3′, R 5′‐GACATGCCGCCTGGAGAAAC‐3′.

All primers were synthesized by Shanghai ShengGong Biotechnology. Quantitative PCR using SYBR Green was performed according to the manufacturer's directions.

### 2.14 Lung histology

Formalin‐fixed, paraffin‐embedded lung tissue sections were examined for airway inflammation with haematoxylin and eosin staining as previously described.^20^, ^21^

### 2.15 Knockdown of Mmp14 or CD100 expression

The following shRNAs were synthesized by Shanghai ShengGong Biotechnology for gene silencing of *Mmp*14: shRNA‐1: 5′‐CCGGCCCTAACTACATACCTTAAATCTCGAGATTTAAGGTATGTAGTTAGGGTTTTTG‐3′, and shRNA‐2: 5′‐CCGGGAGAAGAGCAAACAGACATTTCTCGAGAAATGTCTGTTTGCTCTTCTCTTTTTG‐3′. For CD100: 5′‐CCGGTACTCTGGGACGTCCTATAATCTCGAGATTATAGGACGTCCCAGAGTATTTTTG3′. The shRNA sequences were inserted into the pLKO.1 plasmid between the *Eco*RI and *Nhe*I sites. Scrambled, *Mmp14* shRNA or *CD100*‐shRNA pLKO.1 was co‐transfected with vesicular stomatitis virus (pseudotyping lentiviral vector) into HEK‐293T cells. Viruses were harvested by centrifugation and filtration at 48 h after transfection. All sequences were confirmed by sequencing.

### 2.16 Statistical analysis

Flow cytometric analysis data were analysed using FlowJo software 10.6. Statistical significance was determined by Student's t test or one‐way ANOVA using GraphPad 7.0. *p *< 0.05 was considered statistically significant. Data are expressed as the mean ± standard error of the mean.

## 3 RESULTS

### 3.1 OVA challenge induces airway epithelial cells to yield PLXNB2‐containing exosomes

**FIGURE 1:**
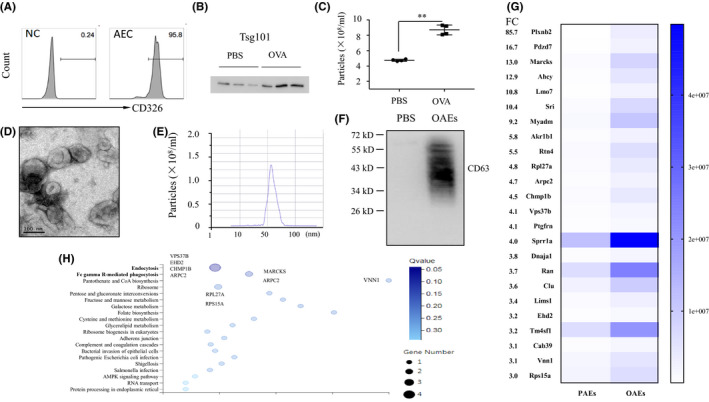
Characterization of OVA‐challenged airway epithelial cell–derived exosomes (OAEs) (A) The purification of primary airway epithelial cells was confirmed by FACS using an anti‐CD326 antibody. (B) Exosomes isolated from the supernatant of 10^6^ primary airway epithelial cells were quantified by WB and confirmed by the common exosomes marker Tsg101. (C) Numbers of exosomes derived from 10^6^ stimulated primary airway epithelial cells under PBS or OVA treatment were quantified by nanoparticle tracking analysis (NTA). **p < 0.01. (D) OAEs from BEAS‐2B cells were imaged by transmission electron microscopy (TEM). (E) A representative graph of ovalbumin‐treated airway epithelial cell–derived exosomes (OAEs) from BEAS‐2B cells analysed by NTA shows a profile of 50–100 nm in diameter. (F) OAEs from BEAS‐2B cells were confirmed by the exosomes marker CD63. (G) Heat map represents differential protein (fold change (FC) ≥3) expression in PBS‐treated airway epithelial cell–derived exosomes (PAEs) and OAEs. The FC was calculated as the iBAQ intensity of OAEs divided by that of PAEs. (H) GO enrichment analysis of the differentially expressed OAE proteins was performed

We first isolated mouse pAECs from the trachea of female C57BL/6J mice, cultured them for 7–10 days and then stained them with an anti‐CD326 antibody to assess their purity, which was confirmed to be approximately 95.8% (Figure 1A). CD63 is considered a common protein in all exosomes independent of their origin and is often used as an exosomal marker. Exosomes from OVA‐challenged pAECs exhibited higher Tsg101 expression than exosomes from PBS‐challenged pAECs, as confirmed by Western blotting (Figure 1B). Using NTA‐based exosome characterization, we found that more particles were present in the medium of OVA‐challenged AECs than in that of PBS‐challenged AECs (Figure 1C). OAEs were evaluated by TEM and NTA, and we found morphology typical of exosomes (Figure 1D), with the diameter of particles in the range of 50–100 nm (Figure 1E). A representative Western blot CD63 band for OAEs is shown in Figure 1F. Using a label‐free proteomic analysis, we found that the composition of protein in OAEs was distinct from that in PAEs. Differential protein expression was defined as a FC for OAEs/PAEs that was ≥3. PlXNB2 was found to be the most enriched protein in OAEs, with a FC of 85.7, which was much higher than that of any other protein (Figure 1G). Based on GO enrichment analysis, the endocytosis‐related proteins VPS37, EHD2, CHMP1B and ARPC2 and the FcγR‐mediated phagocytosis‐related proteins MARCKS and ARPC2 were increased in OAEs compared to PAEs (Figure 1H).

### 3.2 Macrophages produce pro‐inflammatory cytokines and chemokines 16h after internalizing OAEs

**FIGURE 2:**
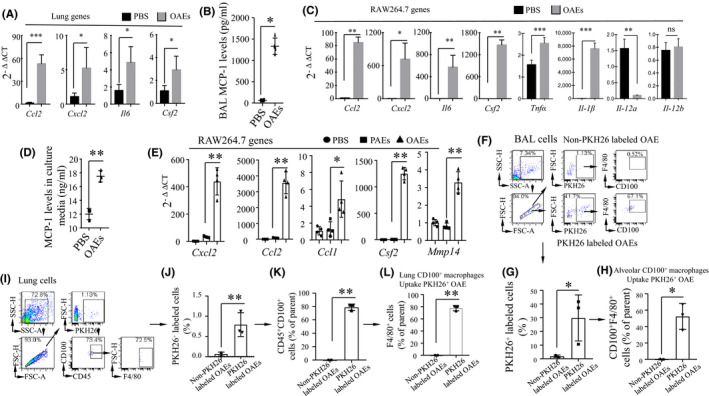
CD100^+^ macrophages internalize OAEs and produce pro‐inflammatory cytokines and chemokines (A) *Ccl2*, *Cxcl2*, *Il6* and *Csf2* transcripts in lungs of PBS‐ or OAE (25 μg)‐treated mice measured by qRT‐PCR (n = 4–6 mice/group). (B) BAL MCP‐1(CCL2) levels in PBS‐ and OAE (10 ng/ml)‐treated mice measured by ELISA. (C) *Ccl2*, *Cxcl2*, *Il6*, *Csf2*, *Tnf*‐*α*, *Il*‐*1β*, *Il*‐*12a and Il*‐*12b* transcripts in PBS‐ and OAE (10 ng/ml)‐treated macrophages measured by qRT‐PCR. (D) The MCP‐1 (CCL2) level in the supernatant of macrophages co‐cultured with PBS or OAEs (10 ng/ml) measured by ELISA. (E) *Ccl2*, *Cxcl2*, *Il6*, *Csf2* and *Mmp14* expression in PBS‐, PAE‐ and OAE (10 ng/ml)‐treated macrophages measured by qRT‐PCR. (F‐H) Flow cytometric analysis of BAL CD100^+^F4/80^+^ macrophages engulfing PKH26‐labelled OAEs. (I‐L) Flow cytometric analysis of lung CD100^+^CD45^+^F4/80^+^ macrophages engulfing PKH26‐labelled OAEs. *p < 0.05, **p < 0.01, *** *p *< 0.005. Values represent the mean ± SD

To test whether OAEs can provoke pro‐inflammatory responses in the lungs, we intranasally challenged mice with a single dose of OAEs. At 24 h, transcription of lung *Cxcl2*, *Ccl2*, *Il6* and *Csf2* was increased in the lungs of OAE‐challenged mice compared to the PBS‐challenged mice (Figure 2A). We also collected BAL fluid to measure MCP‐1 (encoded by *Ccl2*) levels and found that the MCP‐1 transcript was increased in OAE‐challenged mice (Figure 2B). We challenged RAW264.7 macrophages with either OAEs or PBS and found that *Cxcl2*, *Ccl2*, *Il6*, *Csf2*, *Tnf*‐*α* and *Il*‐*1β* transcription increased and that IL‐12a decreased in OAE‐challenged macrophages (Figure 2C). The MCP‐1 levels in the medium of OAE‐challenged macrophages were elevated (Figure 2D). To further compare the pro‐inflammatory properties of OAEs to those of PAEs, we challenged macrophages with PBS, PAEs or OAEs. We found that the transcripts of *Cxcl2*, *Ccl1*/*2*, *Csf2* and *Mmp14* were increased in OAE‐challenged macrophages compared to PBS‐ or PAE‐challenged macrophages. There was no difference in these genes between the PBS‐ and PAE‐challenged macrophages (Figure 2E), suggesting that OAEs are the major driver of inflammatory responses.

PLXNB2, the most enriched protein, is a ligand of CD100 (also named Sema4D). CD100 is highly expressed by immune cells and contributes to the infiltration of pro‐inflammatory cells.^22^, ^23^ Next, we examined whether CD100‐expressing macrophages can engulf fluorescent dye PKH26‐labelled OAEs. We intranasally challenged mice with either unlabelled or PKH26‐labelled OAEs. By flow cytometry analysis, we found that nearly 30% of cells collected from BAL fluid were PKH26^+^ and that approximately 50% of these cells were F4/80^+^CD100^+^ (Figure 2F‐H). Among the lung cells, 0.75% were PKH26^+^, 76% of the PKH26^+^ cells were CD45^+^CD100^+^ and 75% of the CD45^+^CD100^+^ cells were F4/80^+^ macrophages (Figure 2I‐L). These findings suggested that CD100‐expressing macrophages in the airways of the lungs could engulf OAEs and mediate pro‐inflammatory responses.

**FIGURE 3:**
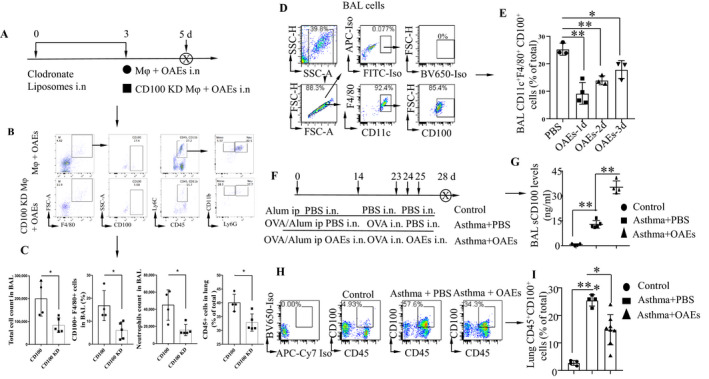
CD100‐expressing macrophages are required for inflammatory cell infiltration, and OAE challenge reduces CD100 expression in BAL macrophages and increases BAL sCD100 levels (A) Protocol for alveolar macrophage depletion, adaptive macrophage transfer and OAEs treatment in C57BL/6 mice. (B) Gating strategy for the BAL cell infiltration in mice described in (A). (C) Flow cytometry analysis of total cell count, CD100^+^ F4/80^+^ macrophages and Ly6G^+^ neutrophils in BAL, and the last plot showed CD45^+^ immune cell percentage in lung. (D‐E) Flow cytometric analysis of CD11c^+^F4/80^+^CD100^+^ cells in the BAL of mice challenged with OAEs on d 1, 2 and 3. (F) Protocol for the OAE‐treated OVA‐induced asthma mouse model. As indicated, mice were sensitized with OVA (100 μg) adjuvanted with Al(OH)3 on d 0 and 14 and challenged with OVA (100 μg) on d 14, 23, 24 and 25. In the asthma +OAEs group, mice were intranasally treated with OAEs (25 μg) in combination with the asthma induction plan. (G) sCD100 levels in the BAL for the three groups listed in (F) measured by ELISA. (H‐I) Flow cytometric analysis of lung CD100^+^CD45^+^ cells in the three groups listed in (F). * *p *< 0.05, **p < 0.01. Values represent the mean ± SD

To determine whether CD100^+^ macrophages play a critical role in airway inflammation, C57BL/6 mice were depleted of macrophages by intratracheal instillation of clodronate liposomes. The efficiency of AM depletion with clodronate liposomes was approximately 80%, as determined by the differential staining of cells collected from BAL fluid and lungs by flow cytometry (Figure S1). Preprepared CD100 knockdown (CD100 KD) or CD100‐expressing macrophages were transferred to alveolar macrophage–depleted mice, followed by OAE treatment. The protocol was described in Figure 3A. The efficiency of CD100 knockdown was confirmed by flow cytometry (Figure S2). The infiltration of inflammatory cells, the expression of CD100 in macrophages and the neutrophil count were assessed by flow cytometry 36 h after OAE treatment, and the gating strategy is shown in Figure 3B. As expected, CD100 KD macrophages resulted in low cell infiltration in both BAL fluid and lungs, as determined by flow cytometry (Figure 3C and Figure S3). In contrast, mice transferred with CD100‐expressing macrophages had high cell infiltration, high CD100 expression and a high neutrophil count in BAL fluid. These data demonstrate that CD100‐expressing macrophages are the critical cells that mediate airway inflammation during OAE exposure.

### 3.3 OAE challenge reduces CD100 expression in macrophages and CD45^+^ immune cells and increases sCD100 levels in the BAL fluid

It has been reported that proteolytic cleavage of membrane‐bound CD100 (mCD100) yields soluble CD100 (sCD100), which promotes pro‐inflammatory responses.^24^, ^25^ To determine whether cleavage of CD100 in AMs can be regulated by OAEs, we intranasally treated mice with OAEs (10 μg/mouse) (PBS as a control) and harvested the BAL fluid on days 1, 2 and 3. Cells in the BAL fluid were collected and analysed by flow cytometry. CD100 expression was reduced on days 1, 2 and 3 in the OAE‐challenged group compared to the PBS‐challenged group (Figure 3D‐E). Furthermore, we intranasally treated OVA‐induced asthmatic mice with OAEs to determine whether OAEs modulate CD100 expression in asthmatic lungs (Figure 3F). We found that BAL sCD100 levels were increased in the OAE‐treated asthma group compared to the PBS‐treated asthma group (Figure 3G). Conversely, CD100 expression in lung CD45^+^CD100^+^ cells was reduced in the OAE‐treated asthma group compared to the PBS‐treated group (Figure 3H‐I). These findings indicated that OAEs might increase sCD100 levels by cleaving membrane‐bound CD100.

### 3.4 OAE‐derived PLXNB2 augments the airway inflammatory response to OVA challenge

**FIGURE 4:**
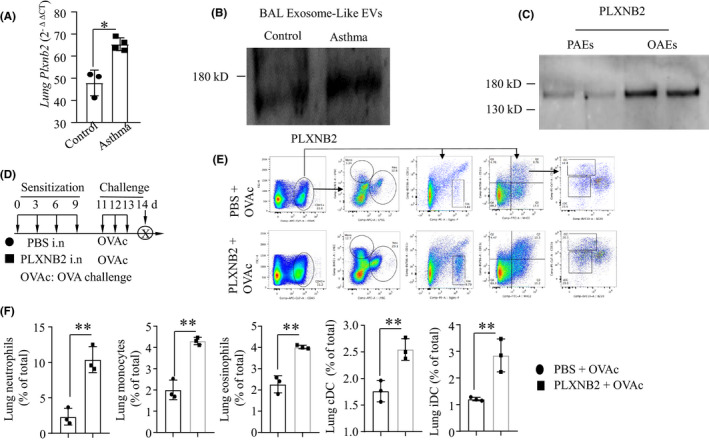
PLXNB2 augments OVA challenge–induced airway inflammation (A) Lung *Plxnb2* transcripts in control and asthma models measured by qRT‐PCR. (B) PLNXB2 protein expression in BAL exosomes isolated from the control and asthma mouse models measured by WB. (C) PLXNB2 expression in PAEs and OAEs measured by WB. (D) The experimental strategy for PLXNB2 sensitization in the OVA‐challenged mouse model. Mice were treated with PBS or recombinant PLXNB2 (300 ng on d 0, 3, 6 and 9; challenged with OVA on d 11, 12 and 13; and killed on d 14. (E) The gating strategy for inflammatory cell analysis in lung by flow cytometry. Monocytes were identified as CD45^+^Ly6C^hi^CD11b^+^; eosinophils were identified as CD45^+^siglecF^+^CD11c^−^; conventional dendritic cells (cDCs) were identified as B220^−^CD11c^+^MHCII^+^CD11b^−^; and inflammatory dendritic cells (iDCs) were identified as B220^−^CD11c^+^MHCII^+^CD11b^+^. (F) Flow cytometric analysis of the effect of PLXNB2 on lung inflammatory cells in OVA‐challenged mouse models. **p < 0.01. Values represent the mean ± SD

The expression of Plxnb2 in asthmatic mouse lungs was higher than that in control lungs (Figure 4A). We isolated exosomes from the BAL fluid of asthmatic or control mice and found that PLXNB2 expression was increased in BAL EXOs from asthmatic mice compared to those from control mice (Figure 4B). To compare the levels of PLXNB2 in PAEs and OAEs isolated from pAECs, Western blotting was performed. We found that the PLXNB2 level was higher in the OAEs than in the PAEs (Figure 4C), which was consistent with the data shown in Figure 1G. To determine the role of PLXNB2 in sensitizing mice to the development of asthma, mice were intranasally treated with either PBS or PLXNB2 (300 ng) on days 0, 3, 6 and 9; challenged with OVA on days 11, 12 and 13; and killed on day 14 (Figure 4D). By flow cytometry analysis, we found that the percentages of lung neutrophils, monocytes, eosinophils, conventional dendritic cells (cDCs) and inflammatory dendritic cells (iDCs) were increased in the mice treated with PLXNB2 compared to those treated with PBS (Figure 4F). These data suggested that PLXNB2 augments the airway inflammatory response. The flow cytometry gating strategy and the representative plots are shown in Figure 4E.

### 3.5 OAEs sensitize mice to asthmatic pro‐inflammatory responses

**FIGURE 5:**
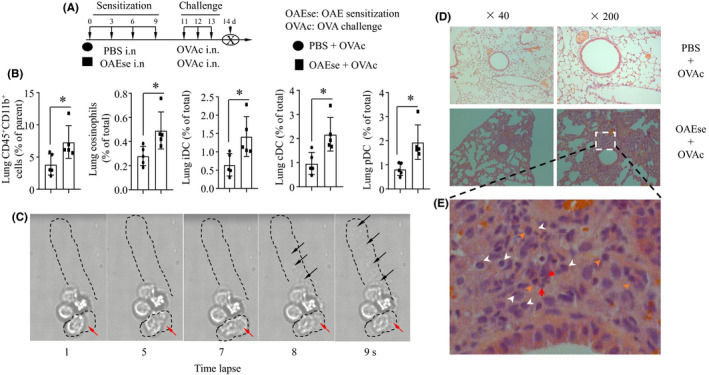
OAE sensitization increases OVA challenge–induced airway inflammation (A) Experimental strategy for OAE sensitization in the OVA‐challenged mouse model. Mice were treated with PBS or OAEs (25 μg) on d 0, 3, 6 and 9; challenged with OVA on d 11, 12 and 13; and killed on d 14. (B) Flow cytometric analysis of the effect of OAE sensitization on lung inflammatory cells in OVA‐challenged mouse models. Plasmacytoid dendritic cells (pDCs) were identified as CD45^+^B220^+^CD11c^+^MHCII^+^CD11b^−^. The labelling strategies for monocytes, eosinophils, iDCs and cDCs were the same as those described in Figure 5E‐F. *p < 0.05. Values represent the mean ± SD. Live image analysis of dynamic changes in eosinophils in response to OAE challenge. Arrows indicate granules released by eosinophils. (D‐E) Representative images of lung pathology in PBS‐sensitized OVA‐challenged and OAE‐sensitized OVA‐challenged mice. Magnification: 40×, 200×. Orange arrows: monocytes; red arrows: eosinophils; and white arrows: neutrophils

To determine whether OAEs would sensitize mice to airway pro‐inflammatory inflammation, mice were intranasally sensitized with either PBS or OAEs on days 0, 3, 6 and 9; challenged with OVA on days 11, 12 and 13; and killed on day 14 (Figure 5A). At 24 h after the final treatment, AHR was assessed. As predicted, OAE sensitization and OVA challenge significantly increased AHR in mice (Figure S4. a‐c, the values are recorded as the mean ± SE. *p < 0.05, **p < 0.01, ***p < 0.001). For both groups, the Re values increased with increasing doses of methacholine. RL showed the same trend as Re, whereas Cdyn showed an opposing trend to that of Re. AHR directly reflects changes in the airway wall structure, which include inflammatory cell infiltration. Consistent with this, we observed that OAE‐sensitized and subsequently challenged mice had inflammatory cell infiltration in the peribronchial and perivascular areas (Figure 5D). By flow cytometric analysis, we found that OAE sensitization increased the percentages of lung CD45^+^CD11b^+^ cells, eosinophils, iDCs, cDCs and plasmacytoid dendritic cells (pDCs) (Figure 5B, Figure S5). Since eosinophils are the key cells in the development of asthma, we observed the responses of eosinophils to OAE challenge with a live‐cell workstation. The dynamic changes in the morphology and movement (red arrow) of eosinophils were recorded. We found that the amoeboid movement and degranulation (ejection of granules) of eosinophils were increased in response to OAE challenge (Figure 5C). The histopathology of the lungs showed destruction of the lung structure and accumulation of inflammatory cells, including neutrophils (white arrows), eosinophils (red arrows) and monocytes (orange arrows), in the OAEs +OVA group (Figure 5E). Our data indicated that sensitization with OAEs could amplify the lung pro‐inflammatory response in OVA‐induced asthmatic lungs.

### 3.6 OAEs induce the cleavage of CD100 in macrophages via MMP14

**FIGURE 6:**
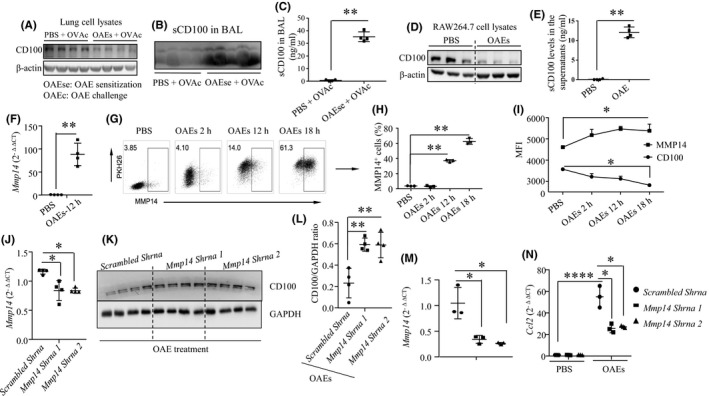
OAEs induce cleavage of CD100 in macrophages via MMP14. (A) CD100 protein expression in lung cell lysates from PBS +OVAc (c: challenge) and OAEse (se: sensitization) + OVAc (c: challenge) mice quantified by WB. (B) BAL protein of each mouse was extracted, and the quantity of CD100 was imaged by WB. (C) sCD100 levels in the BAL collected from the PBS +OVAc and OAEse +OVAc mice quantified by ELISA. (D‐E) CD100 and sCD100 levels in PBS‐ and OAE‐treated macrophages quantified by WB and ELISA. (F) *Mmp14* levels in PBS‐ and OAE‐treated macrophages quantified by qRT‐PCR. (G‐I) Flow cytometric analysis of MMP14 and CD100 expression in PBS‐challenged and PKH26‐labelled OAE‐challenged macrophages at different time points. (J) *Mmp14* expression in scrambled shRNA‐ and *Mmp14* shRNA‐treated macrophages. (K‐L) Change in CD100 expression (upper band) in scrambled shRNA‐ and *Mmp14* shRNA‐treated macrophages treated with OAEs. (M‐N) Change in *Ccl2* expression in scrambled shRNA‐ and *Mmp14* shRNA‐treated macrophages treated with PBS or OAEs. *p < 0.05, **p < 0.01. Values represent the mean ± SD

To determine whether OAEs can induce the cleavage of CD100, we assessed CD100 expression in lung cell lysates and sCD100 levels in the BAL from PBS‐ and OAE‐sensitized mouse models (experimental strategy is presented in Figure 5A) by Western blotting and ELISA. CD100 expression in the lung homogenates in the OAE‐sensitized group was reduced (Figure 6A), coinciding with an increase in sCD100 expression in the BAL fluid (Figure 6B‐C). These findings indicated that OAEs might induce the cleavage of CD100. Next, we challenged macrophages with either PBS or OAEs and detected CD100 changes. We found that CD100 expression in cell lysates was reduced (Figure 6D), whereas sCD100 levels were higher in the OAE‐sensitized group than in the PBS‐treated group (Figure 6E). MMP14 (MT1‐MMP) has the ability to cleave mCD100.^26^ To examine whether MMP14 is involved in the proteolytic cleavage of CD100 in response to OAE challenge, we first measured *Mmp14* expression and observed that *Mmp14* expression was significantly increased in OAE‐challenged macrophages compared to PBS‐treated macrophages (Figure 6F). We also observed a time‐dependent elevation in MMP14 expression in OAE‐challenged macrophages, as confirmed by flow cytometry (Figure 6G‐I), accompanied by a gradual decline in CD100 expression (Figure 6I), suggesting that cleavage of CD100 occurred after OAE stimulation. We further confirmed the role of MMP14 in CD100 cleavage by knocking down *Mmp14* expression in macrophages (Figure 6J). In co‐culture with OAEs, *Mmp14* knockdown increased CD100 expression in macrophages (Figure 6K‐L). In another experiment, we found that knockdown of Mmp14 expression (Figure 6M) suppressed the transcription of *Ccl2*, *Ccl5* and *Cxcl2* (Figure 6N, Figure S6) in OAE‐challenged macrophages. These findings indicate that MMP14 is able to cleave CD100 and thereby mediates the expression of pro‐inflammatory cytokines in macrophages challenged by OAEs.

## 4 DISCUSSION

Exosomes are secreted by a variety of cells and provide an efficient mediator for intracellular communication with innate immune cells, especially for structural cells with a low potency to produce cytokines and chemokines. In this study, we identified OAEs as potent elements that increase the AHR and induce the infiltration or activation of macrophages, neutrophils and eosinophils in the airways. We also clarified that OAEs could trigger proteolytic cleavage of CD100 in macrophages and promote downstream pro‐inflammatory responses in the airways. These observations support our hypothesis that AECs can regulate innate immune cells, especially macrophages, via exosomes^27^, ^28^, ^29^ to influence the development of asthma.

It is well known that the Th2 response plays a dominant role in the OVA/alum‐induced mouse asthma model. However, asthma is a complex disease in the clinic and involves a variety of cells. Pulmonary macrophages are the most abundant innate immune cells in the resting lungs and orchestrate the initiation and resolution of the immune response in the lungs.^30^ Recently, an increasing number of studies have suggested that lung macrophages are involved in the pathogenesis of asthma.^31^ For example, a very high percentage of macrophages was found in human BAL cells collected from severe asthma patients.^32^ BAL macrophages with impaired efferocytotic capacity could result in neutrophil persistence and contribute to the neutrophilic asthma phenotype.^33^ In this study, we found increased infiltration of neutrophils and monocytes in the lungs when OAEs were overlaid in the airway in an OVA/alum‐induced asthma model. This phenomenon is different from the classic phenotype but is close to the clinical severe asthma phenotype. Further experiments identified macrophages as the main recipient cells of OAEs in the lungs and BAL fluid, and OAE‐activated macrophages displayed enhanced MMP14 expression and CD100 cleavage and increased cytokine production. Therefore, we confirmed that macrophages are involved in OAE‐regulated airway inflammation.

Cytokines and chemokines play important roles in the development of airway inflammation. Macrophages have a potent ability to produce cytokines and chemokines. It has been reported that CXCL2^34^ and IL‐6^35^ levels are increased in patients with severe steroid‐resistant asthma compared to those with mild or moderate asthma, occurring independent of type 2 inflammation. Our asthma+OAE model has a phenotype that is similar to that of severe steroid‐resistant asthma, which is characterized by neutrophil infiltration. We found that OAE‐activated macrophages produced MCP‐1, CXCL2, IL‐6, GM‐CSF, TNF‐α and IL‐1β, which are well‐recognized cytokines and chemokines with pro‐inflammatory functions during airway inflammation.

Exosomes were found to be able to initiate and propagate lung inflammation a decade ago.^36^ One study showed that exosomes from OVA‐pulsed dendritic cells could efficiently induce antigen‐specific CD8^+^T cells.^37^ In particular, IL‐13‐challenged epithelial cell–derived exosomes can enhance the proliferation and chemotaxis of undifferentiated macrophages in the lungs under inflammatory asthmatic conditions.^2^ The novelty of our study is that OVA‐pulsed epithelial cell–derived exosomes could augment airway inflammation by activating macrophages via cleavage of CD100 by MMP14. These findings have deepened our understanding of the communication between AECs and macrophages during asthma.

Many factors can affect the biogenesis and release of exosomes. We have demonstrated that OVA challenge in AECs can increase the release of exosomes and that the underlying mechanisms are known. By GO enrichment analysis, we found that the levels of the endocytosis‐related proteins VPS37, EHD2, CHMP1B and ARPC2 were increased in OAEs. Vacuolar protein sorting–associated protein 37B (VPS37B) is a component of ESCRT‐1, which mediates endosomal sorting.^38^ Charged multivesicular body protein 1B (CHMP1B) also participates in the process of multivesicular body (MVB) formation and vesicular trafficking.^39^ EHD2 and ARPC2 play roles in membrane trafficking between the plasma membrane and endosomes.^40^, ^41^ The roles in mediating OAE formation and release require further investigation.

Furthermore, OAEs contain the phagocytosis‐related proteins MARCKS^42^ and ARPC2,^43^, ^44^ which might facilitate macrophage phagocytosis of OAEs. Indeed, we found that CD100‐expressing macrophages could engulf OAEs, augmenting the production of pro‐inflammatory cytokines and chemokines. One study showed that high glucose–treated macrophage‐derived EXO‐challenged macrophages could secrete relatively high levels of related inflammatory molecules and promote NF‐κB p65 signalling activity.^45^ Exosomes released by ER‐stressed HepG2 cells were found to significantly enhance the expression levels of several cytokines, including IL‐6, MCP‐1, IL‐10 and TNF‐α, in macrophages.^46^ The findings from other studies support our hypothesis that macrophages phagocytose OAEs and facilitate pro‐inflammatory responses.

Importantly, OAE‐engulfing macrophages expressed high levels of MMP14, which could proteolytically cleave CD100 into sCD100. MMP14 is recognized as a prominent member of the MMP family, as it has broad substrate specificity and is required for the activation of other MMPs, such as MMP2^47^ and MMP13.^26^ Deletion of MMP14 results in fatal lung dysplasia.^48^ There are a few reports about CD100 shedding via MMP14. MMP14 mediates CD100 shedding and promotes CD8+ T cell functions.^49^ MMP14 controls tumour‐induced angiogenesis through the release of CD100.^50^ Our findings demonstrate that the OAE‐induced MMP14‐triggered cleavage of CD100 in macrophages contributes to airway inflammation.

Numerous studies have shown that monocytes stimulated with sCD100 produce the pro‐inflammatory cytokines IL‐6, IL‐8 and TNF‐α^51^ and that sCD100 also augments infection and phagocytosis.^52^ By binding to PLXNB2, soluble CD100 promotes the production of CXCL‐1, CCL‐20, IL‐1β and IL‐18 by keratinocytes and activates the NLRP3 inflammasome. PLXNB2 is overexpressed in lesional skin in psoriasis and can promote skin inflammation.^53^ Plexin B2 and CD100 act as adhesion molecules involved in monocyte‐endothelial cell binding.^54^ Our investigation demonstrated that OAEs (containing PLXNB2) or exogenous PLXNB2 sensitization could augment or propagate OVA challenge–induced airway inflammation.

In summary, AECs exhibited an enhanced ability to produce exosomes in response to OVA challenge. The OAEs could enhance neutrophilic and monocytic airway inflammation by activating macrophages via upregulation of MMP14 expression and cleavage of CD100. Therefore, the OAE‐induced cleavage of CD100 by MMP14 in macrophages is a novel therapeutic target for treating asthma.

## CONFLICT OF INTEREST

The authors declare that they have no conflict of interest.

## AUTHOR CONTRIBUTIONS

**Yi Yu:** Data curation (lead); Formal analysis (lead); Investigation (equal); Methodology (equal); Writing‐original draft (equal). **Yao Zhou:** Data curation (supporting); Formal analysis (equal); Investigation (equal); Methodology (equal); Writing‐original draft (supporting). **Caixia Di:** Conceptualization (equal); Data curation (equal). **Caiqi Zhao:** Investigation (supporting); Methodology (equal). **Jie Chen:** Investigation (supporting); Methodology (equal). **Wen Su:** Formal analysis (equal). **Qun Wu:** Formal analysis (equal). **Min Wu:** Conceptualization (equal); Project administration (equal); Writing‐review & editing (equal). **Xiao Su:** Conceptualization (equal); Data curation (equal); Formal analysis (equal); Funding acquisition (equal); Project administration (equal); Validation (equal); Writing‐review & editing (equal). **Zhenwei Xia:** Conceptualization (lead); Data curation (equal); Formal analysis (equal); Funding acquisition (equal); Project administration (equal); Validation (equal); Writing‐review & editing (equal).

## Supporting information

Figure S1‐S6

## Data Availability

The data that support the findings of this study are available from the corresponding author upon reasonable request.
