# Supplementary material for: Increased airway epithelial cell–derived exosomes activate macrophage‐mediated allergic inflammation via CD100 shedding
Source: J Cell Mol Med. 2021 Aug 20;25(18):8850–62. doi: 10.1111/jcmm.16843 (PMC8435458; doi:10.1111/jcmm.16843)
Supplement: Supplementary file 1 — Figure S1‐S6 [file JCMM-25-8850-s001.pdf]

Figure S1

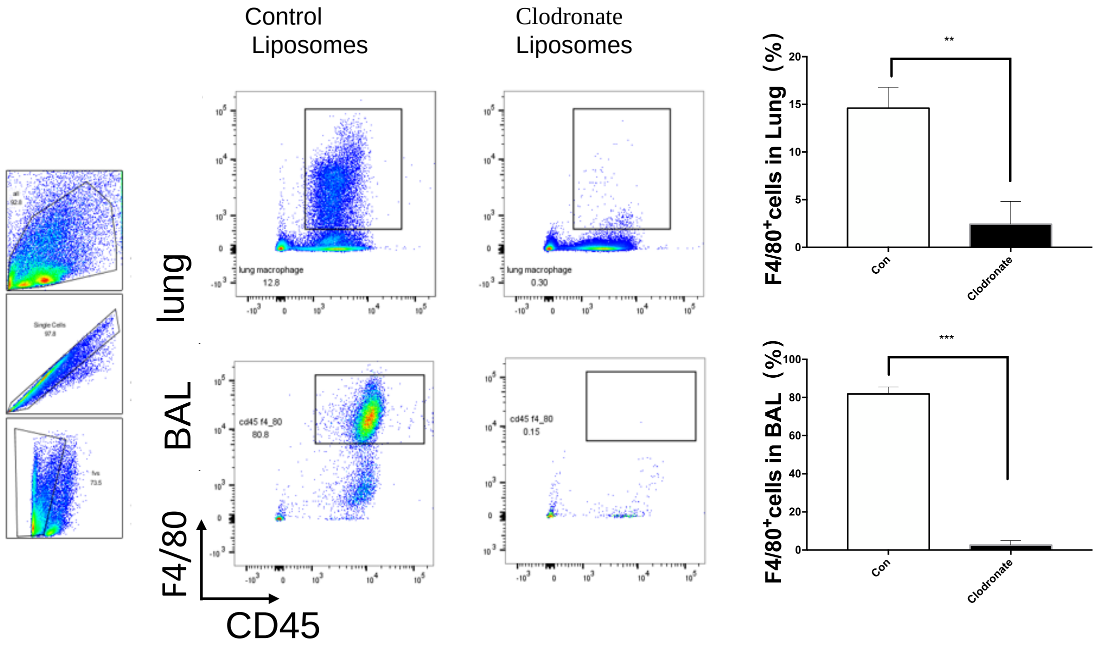

S1 Depletion of alveolar macrophages with clodronate liposomes. Mice received one dose of clodronate as described in the method, and were sacrificed 72h later. BAL and lungs were collected and single cell suspensions were evaluated by flow cytometry. The left panel shows gating strategy, and the right penal shows percentage of F4/80 positive cells in lung and in BAL, confirming that AM are depleted.

Figure S2

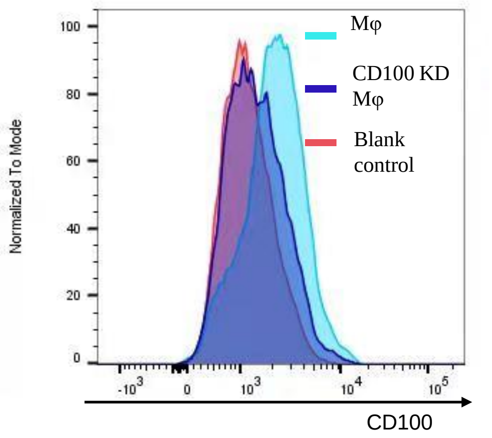

S2 Mean fluorescence intensity of CD100. shRNAs were used for gene silencing of CD100 as described in the method. CD100 knock down RAW264.7 (CD100 KD Mφ) showed decreased MFI.

Figure S3

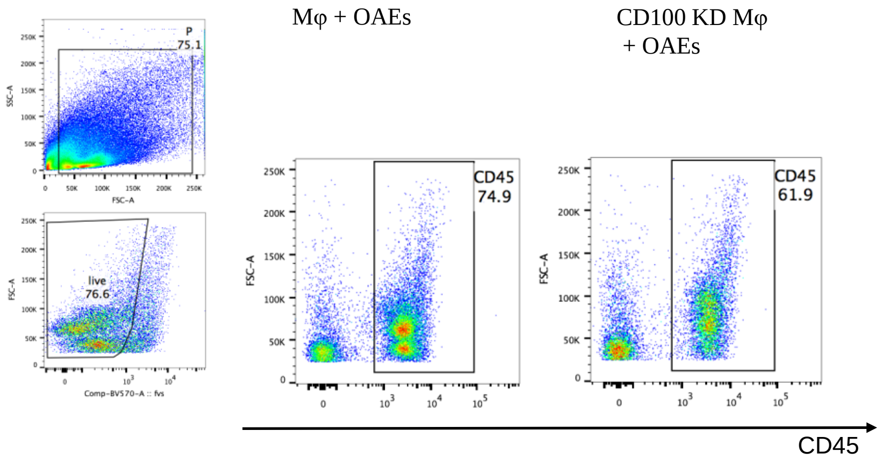

S3 FACS gating strategy and the presentative plots of immune cells in the lung of mice for Fig 3C

Figure S4

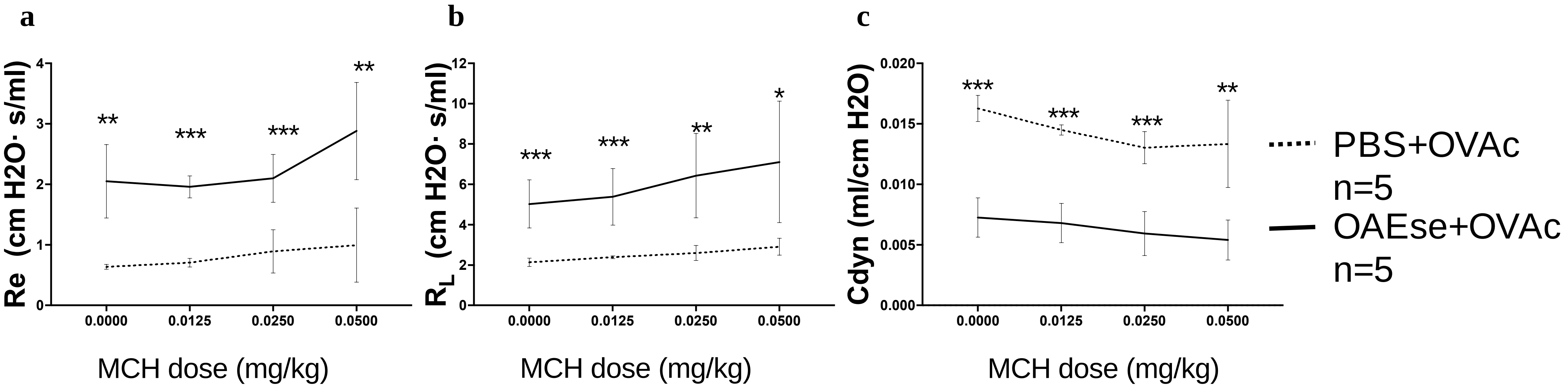

S4 Airway hyper-responsiveness (AHR). a) The resistance of expiration (Re) b)The resistance of the lung (RL) c) The respiratory dynamic compliance (Cdyn) were recorded to evaluate the reaction of mice to a methacholine chloride gradient (0.0125, 0.025, 0.05, mg/kg body weight). The values are recorded as the mean ± SD. **p* < 0.05, ***p* < 0.01, ****p* < 0.001). Increased AHR were confirmed in OAEse+OVAc mice.

Figure S5

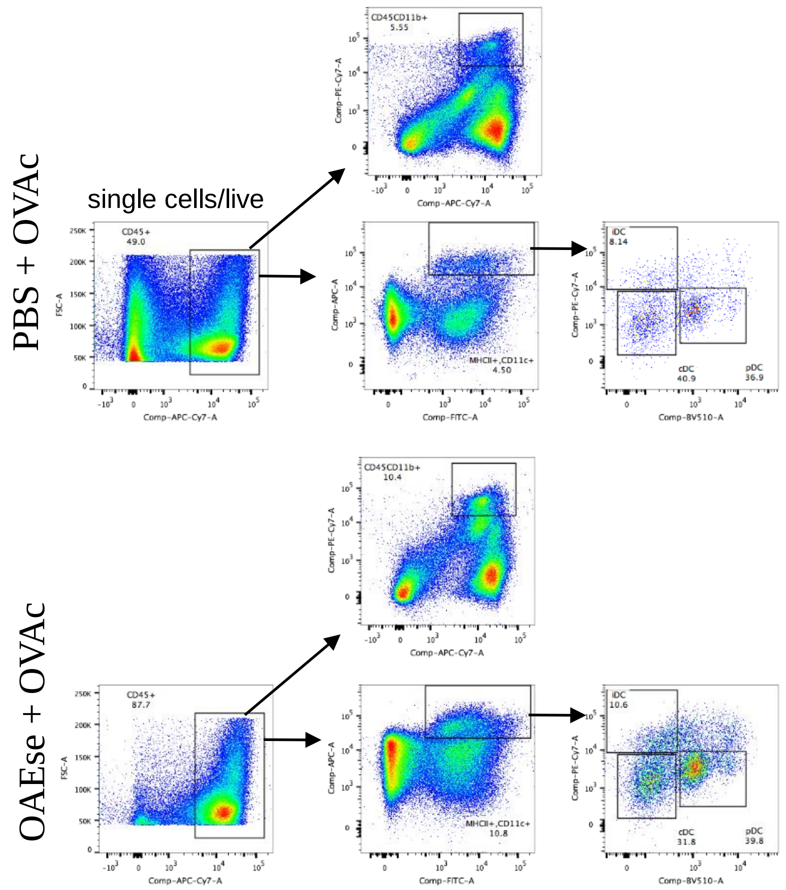

S5 Flow cytometry gating strategy and the presentative plots in the lung of mice for Fig 5B

Figure S6

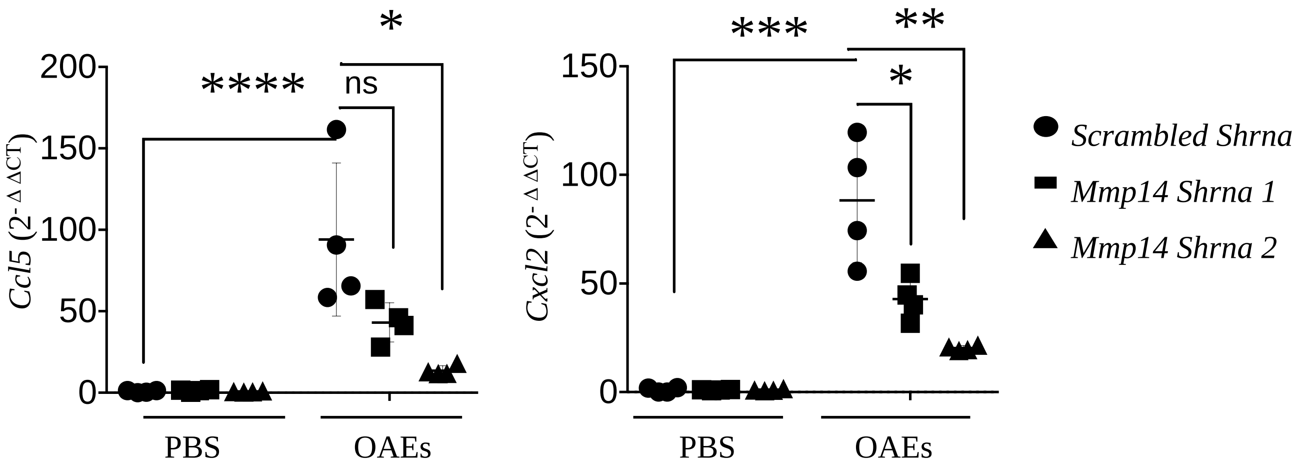

S6 Change in *Ccl5* and *Cxcl2* expression in scrambled shRNA- and *Mmp14* shRNA-treated macrophages treated with PBS or OAEs. *P<0.05, **P<0.01， *** P<0.001， ****P<0.0001. Values represent the mean ± SD.
